# Development of an Optical Gas Leak Sensor for Detecting Ethylene, Dimethyl Ether and Methane

**DOI:** 10.3390/s130404157

**Published:** 2013-03-28

**Authors:** Qiulin Tan, Xiangdong Pei, Simin Zhu, Dong Sun, Jun Liu, Chenyang Xue, Ting Liang, Wendong Zhang, Jijun Xiong

**Affiliations:** 1 Key Laboratory of Instrumentation Science & Dynamic Measurement, North University of China, Ministry of Education, Taiyuan 030051, Shanxi, China; E-Mails: qltan99@163.com (Q.T.); 18234138669@163.com (X.P.); 18734856312@163.com (D.S.); liuj@nuc.edu.cn (J.L.);18234109017@163.com (C.X.);wdzhang@nuc.edu.cn (W.Z.); 2 National Key Laboratory for Electronic Measurement Technology, Taiyuan 030051, Shanxi, China; E-Mails: zsm19870505@163.com (S.Z.);15209213398@163.com (T.L.); 3 Department of Mechanical and Biomedical Engineering, City University of Hong Kong, Tat Chee Avenue, Kowloon, Hong Kong

**Keywords:** optical gas leak sensor, IR absorption spectrum, ethylene, dimethyl ether, methane

## Abstract

In this paper, we present an approach to develop an optical gas leak sensor that can be used to measure ethylene, dimethyl ether, and methane. The sensor is designed based on the principles of IR absorption spectrum detection, and comprises two crossed elliptical surfaces with a folded reflection-type optical path. We first analyze the optical path and the use of this structure to design a miniature gas sensor. The proposed sensor includes two detectors (one to acquire the reference signal and the other for the response signal), the light source, and the filter, all of which are integrated in a miniature gold-plated chamber. We also designed a signal detection device to extract the sensor signal and a microprocessor to calculate and control the entire process. The produced sensor prototype had an accuracy of ±0.05%. Experiments which simulate the transportation of hazardous chemicals demonstrated that the developed sensor exhibited a good dynamic response and adequately met technical requirements.

## 1. Introduction

Gas leaks are a serious threat to equipment reliability and safety, and considerable effort has been invested over the last few decades in designing gas leak detection techniques [1–3]. The current sensors generally offer short-lives, poor stability [4], short cycle regulation and other issues. Specific performance problems are as follows [5–8]:

(1) Short lifetime of the detection device. Due to the poor performance of existing detection elements, the average usage lifetime is only 3–6 months, so if a component's activity decreases without being detected in time, the ranging precision is decreased, or it will became a dead probe, which simply does not react to a gas.
(2) Sensor accuracy and linearity of product certification rules of order do not match the basic error range, indicating the error between the actual values is larger or lower than the standard, the high-end is closed, and low end errors are not allowed.
(3) With the unstable performance, the digital display bounces. Some components suffering from the assault of high concentrations of gas in the measurement range would become invalid within a few minutes. Some components do not lose activity, but the device sensitivity decreases significantly. When the sensor suffers from vibration (such as during blasting or mobile collisions) and wind speed changes, the digits bounce and zero drift emerges, thus resulting in job insecurity [9].

Optical gas detection that is based on the IR absorption spectrum, which currently occupies an important place in the gas detection technical field, is superior to traditional detection methods (for example, chemical reactions) in terms of accuracy, speed, and even security. It commonly involves the use of infrared (IR) optical detection instruments that are simple, durable, and reliable [10,11]. The gas molecules absorb IR radiation so their presence can be detected using IR spectroscopy [12]. IR technology has a variety of applications [13]. Furthermore, the selectivity of an IR sensor is high, and its range of sensitivity is large (from a few parts per million to 100% concentration) [14].

A gas sensor needs to be adjusted weekly, and frequent adjustments to the movement sensor will affect the moisture sensor instrument's performance and lifetime. Therefore, it is necessary to develop high-performance gas sensors to solve the above shortcomings. Infrared sensors involve the use of a gas absorption-type gas sensing principle, so their performance is completely different from the conventional gas sensors, and some of their unique advantages are as follows:

- Fast response: no delay due to the chemical reactions process;
- Good anti-poisoning performance: single frequency spectrum offers selectivity;
- Stability: the device itself does not participate in any chemical reactions. Compared with chemical gas sensor, infrared sensors can overcome the fatal weakness of the former, sensor surface carbonization;
- Dynamic range, theoretically from 0 to 100% full range of measurement;
- Affected by temperature, humidity, dust and other environmental factors;
- Offer long-term stability, so routine maintenance is easy.

Because of infrared gas sensors' many unique advantages [15], they are especially suited for harsh and hazardous environments applications. Currently several infrared gas sensor types have been studied and there has been significant progress in this field.

## 2. Principles

When a beam of IR radiation containing a range of wavelengths is incident upon a material, the material absorbs certain wavelengths [16]. Even if the strength of the radiation is insufficient to cause an electronic energy transition [17], it can still cause vibrational and rotational transitions. The spectroscopic technique based on these transitions is called molecular vibration spectroscopy. If the energy of the electromagnetic wave equals the molecular energy level of the material [18], the electromagnetic wave can be absorbed, thereby causing an electronic state transition. Thus, IR irradiation may cause a molecular vibration energy level transition as long as condition (1) is satisfied:

(1) $${\text{E}}_{\text{IR}}=\Delta {\text{E}}_{\text{molecular vibration}}$$

**Figure 1.**
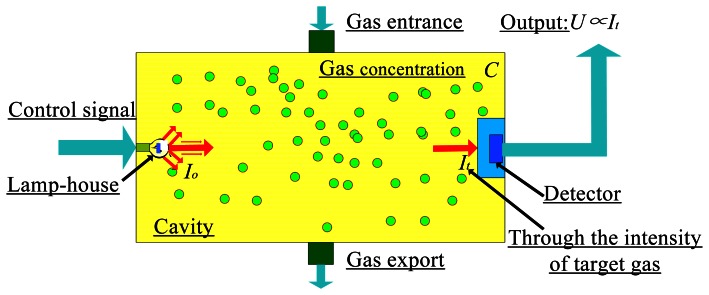
Gas detection model based on IR absorption spectroscopy.

Analyses show that molecules such as methane, ethylene, and dimethyl ether absorb specific wavelengths in the IR spectrum [19]. Therefore, as shown in Figure 1, the molecular vibration spectroscopy with IR irradiation method can be used to detect gas leakage.

Here we investigate the relationship of certain variables to the input and output. An IR light source emits radiation at intensity ***I****_o_*, which is absorbed by a gas of concentration ***C*** and detected as intensity ***I****_t_* by the detector. First, the intensity signals weakened by the gas molecules can be directed into a detector of ***I*** (IR absorption intensity) in Figure 1. Secondly, we consider the relationship between ***C*** (gas concentration) and the intensity of the light emitted by the IR light source, ***I****_o_*, with the weakened light intensity, ***I****_t_*.

A filter is an element which changes the spectral distribution, vibration direction and intensity of radiant energy that reaches the detector in a determined way, and can be divided into neutral filters and interference filters. Wherein the neutral filter can attenuate the power of all the wavelengths reaching the detector by the same multiple within a predetermined wavelength range; interference filters enable the desired the light in a certain wavelength range pass through or reflect using one or several layers of transparent film whose thickness and wavelengths show certain relations to generate interference. As to the applications of the infrared detectors, filters must meet the following two conditions:

(1) According to the specific requirements of the application, in the ultraviolet to the far infrared region they must define the spectral absorption region of the sensitive components;
(2) To achieve an optical interface between the sensitive element and the external environment, while able to achieve a reliable sealing performance.

Combined the infrared detection basic principle with gas detection model assumptions, we selected interference filters, through which the sensitive element performs a selective absorption of infrared radiation, but also eliminates the spectral influence of other irrelevant wavelength regions conductively. Narrow band pass optical filters adapted to infrared detectors are usually prepared on a silicon or germanium substrate, which is made of a crystalline material such as: BaF_2_, CaF_2_, KBr, *etc.* [20]. These filters have an extremely narrow spectrum of performance, and among them, those produced by InfraTec (Dresden, Germany) are superior to others. In our research, we selected their HC series, specifically made of CaF_2_. Some useful specific parameters of gas detection filters are shown in Table 1.

**Figure 2.**
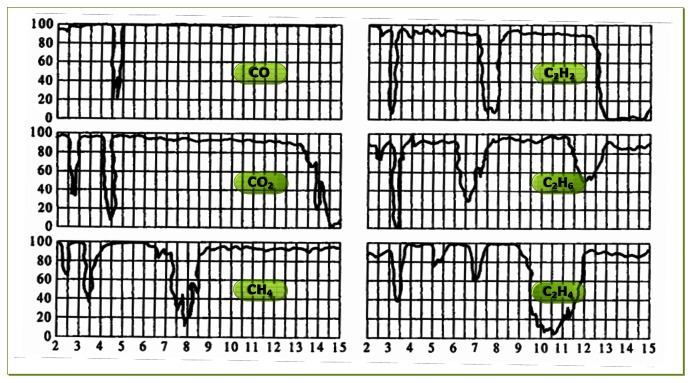
Infrared transmission spectrum of several typical gases.

As to the choice of the parameters of the filter chip, we need to determine them according to the requirements of the intended detection application. For gas detection applications, it is necessary to analyze the infrared absorption spectrum of the detected gas. According to the absorption characteristics of the gas, we can select an optimal filter, and filter out infrared radiation of the other regions, Thus we avoid the impact that the infrared radiation of the other spectral regions being absorbed by the sensitive element. Infrared transmission spectra of several typical gases are shown in Figure 2.

**Figure 3.**
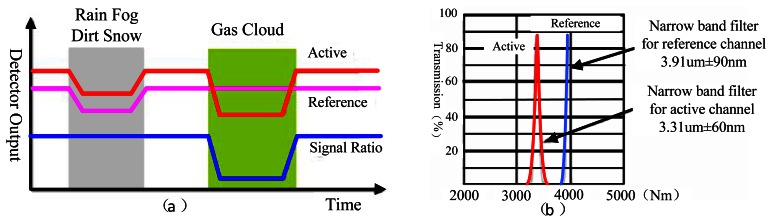
(**a**) The relationship of response channel, reference channel output signal and the input signal; (**b**) Optical performance of narrowband filter.

The dual channel double wavelength detection method takes two output signal channels from the ratio of concentration calculation scheme, with the aim of eliminating the interference from the outside environment. Figure 3(a) shows the relationship of the response channel, reference channel output signal and the input. In our research, we mainly focus on the gas detection of ethylene, dimethyl ether and methane. According to the analysis of their FTIR spectra, there is a special absorption peak near the characteristic absorption peak of methane (3.31 μm), so the parameters of the selected filter sheet as shown in Figure 3 (b). The optics filter parameter is 3.31 μm ± 60 nm in the gas examination response wavelength region, but a 3.91 μm ± 90 nm reference channel is used for gas examination. Regarding other gas examinations we may act according to the above method. The parameter selection principle is that the reference channel for most gases must not have an absorption peak in this region.

Through analysis of the infrared detection principle and dual wavelength ratio method, the paper introduces the reference detection wavelength, the purpose is to eliminate the intensity of light and external environment factors, and when the information is integrated many gas concentration detection schemes can be designed and implemented [21]. The method involves increasing its corresponding gas concentration measurement with the corresponding infrared detector, and sharing the reference detector to achieve multiple gas measurement. In the experimental process design, at present the methane and carbon dioxide gas sensor, methane and carbon monoxide gas sensors and carbon dioxide and ethylene sensor research were successfully realized, through solving the problem of light intensity and the performance of the detector with further improvements should allow three and more gas measurements with a sensor. One only needs to replace the corresponding filter sheet corresponding to the target gas, without changing the structure of the sensor, for the sensor to detect a variety of gases, so it has the versatility of detecting leaks of the three gases.

The double-detector structure we employ here involves a reference detector and a working detector. The convergence of rays of the IR source is reflected to the detector. The working detector has a filter for the target gas, whereas the reference detector has a filter for a different wavelength. That is, the working detector is used to detect the target gas while the reference detector is used to eliminate effects not due to the target gas. In practice, the reference detector provides the baseline value or zero, whereas the working detector provides the signal. The difference between the detectors is the actual measurement. This arrangement compensates for variations in detector sensitivity that occurs with the passage of time [22]. For example, the intensity of the light source changes with time, contributing to an offset. The dual detector structure layout minimizes this type of shift. Moreover, the optical path length in this configuration is double that of other layouts, producing a signal of high strength [23].

**Figure 4.**
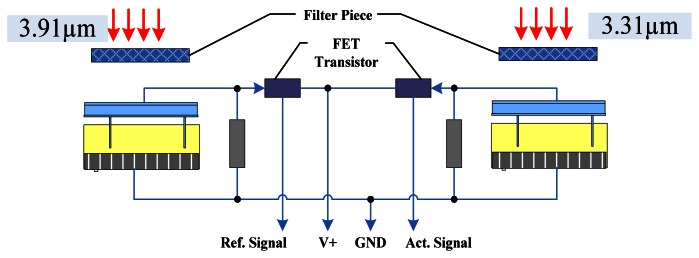
Schematic diagram of IR gas sensor.

To detect gas leakage, we focus on a very narrow region of the IR spectrum [24]. Other wavelengths of light are filtered out before reaching the surface of the detector. Figure 4 shows a schematic of the IR gas sensor [25].

We add another IR detector to detect the original IR light intensity ***I****_0_* before gas absorption. We use another detector that senses an extremely narrow band of IR wavelengths because the IR light emitted from the light source is assumed to have a continuous spectrum (*i.e.*, the light intensity at each wavelength is the same). The intensity of the light at these wavelengths within the chamber does not change; therefore, the light intensity detected in the chamber by this sensor is ***I****_0_*.

We refer to the above-described method of detection as dual-wavelength IR absorption. A model that explains the method is given below. According to the Lambert-Beer law [26]:

(2) $$I=I_{0}\;\text{exp}(-KC^{n}L)$$

where ***I*** and ***I****_O_*, correspond to the light at the response channel and reference channel, respectively; ***K*** and ***L*** are constants denoting the scale factor and gas incident process, respectively; and ***n*** is a correction constant.

(3) $$\begin{array}{c} \{\begin{array}{lll} \text{Act}. & & U_{t}\propto I_{t}\;;\\ \text{Re}\;f. & & U_{o}\propto I_{o}\;;\\ \text{Absorption Law}: & : & C\propto (I_{o},I_{t}). \end{array} \end{array}$$

(4) $$F_{a}=\frac{U_{\text{Ref}}-U_{\text{Act}}}{U_{\text{Ref}}}=\frac{I_{\text{Ref}}-I_{\text{Act}}}{I_{\text{Ref}}}$$

Furthermore, where the detector output signal corresponding to ***I_0_*** is ***U_O_***, which is defined as the reference signal (***Ref***.); the detector output signal corresponding to ***I_t_*** is ***U_t_***, which is defined as the response signal (***Act***.); and ***F_a_*** is the relative absorption rate.

## 3. Sensor Design

**Figure 5.**
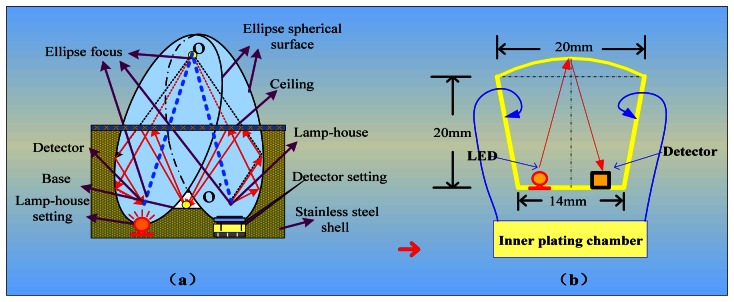
(**a**) Gas chamber optical path and (**b**) diagram of chamber structure.

We integrated the components of the IR detector to make it more accurate, convenient and reliable. We used detectors for wavelengths of 3.31 μm and 3.91 μm, an IR light source, a circuit board, and a metal net integrated into a gold-plated chamber. The porous gold-plated metal and the metal net allow gas to diffuse into the gas chamber and eliminate the influence of the external environment. Figure 5(a) shows the gas chamber and the optical path.

In the interest of having a long light path, high degree of convergence and a simple detector and light source integration technology in the small volume, this structure ensures a long optical path with a smaller air chamber, which enables easier gas exchange with the external environment. At the same time, a smaller gas chamber that has five-fold reflection optical paths can accommodate the signal pre-processing circuit because the volume of the sensor is reduced. The design of the air chamber structure is shown in Figure 5(b). The outer shell of the sensor is made of stainless steel. Two detectors and an IR light source are connected to a circuit board and installed in the gas chamber. A layer of filter membrane that protects the detectors from dust and moisture is embedded in the casing and the interior casing of the sensor. Diaphragm filters were interbedded on the inner membrane, and a small hole is used to exchange gas with the external environment. In one side of the outer shell is gas, whereas the other side is closed; the gas chamber is contained in the outer shell. Filtration is performed by a hydrophobic micro-porous filtration membrane with a pore diameter in the range of 0.2 to 3 μm.

**Figure 6.**
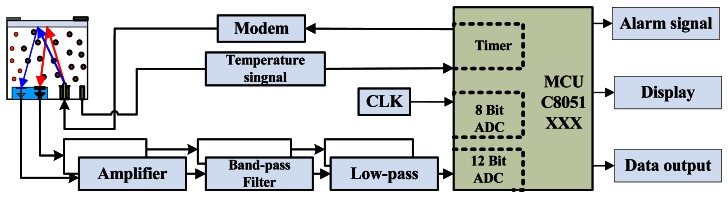
Schematic diagram of the detection system.

To detect gases on the basis of their absorption spectra, we designed a weak-signal detection circuit comprising a preamplifier, a filter, an A/D converter, and a liquefied crystal display. The output signal is pre-processed and amplified, converted to digital form, processed, and ultimately classified by the micro-controller. For miniaturization and portability, a highly integrated hybrid micro-controller unit (MCU) is used, as shown in Figure 6. The detection system includes an IR gas sensor, a signal processing circuit, a light source modulation circuit, an MCU, and an external data transmission and alarm device.

**Figure 7.**
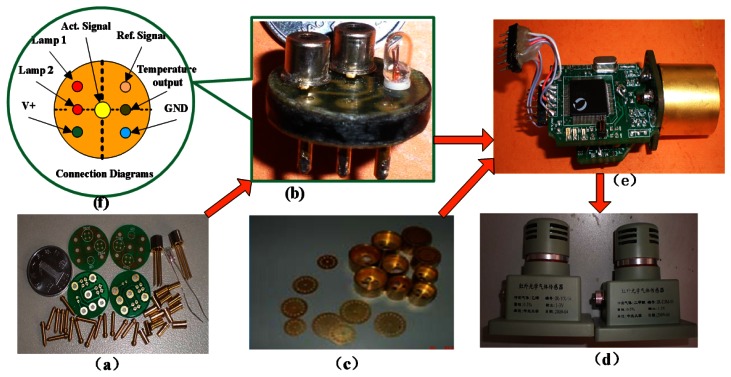
(**a**) Basic components of sensor; (**b**) integrated detector and light source; (**c**) miniature gilded air chamber; (**d**) peripheral IC of sensor; (**e**) end-product; and (**f**)the output interface of the detector.

The signal processing circuit detects and amplifies the weak sensor signal. The MCU mainly performs calculations and controls the other components. The module is easy to use and can also be used to detect other gases if the sensitive probe is replaced. Figure 7 shows the basic components and final sensor.

## 4. Experiments

**Figure 8.**
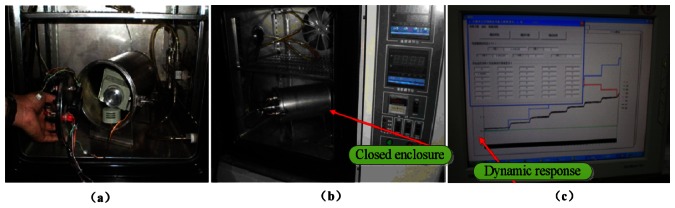
(**a**,**b**) The sealed chamber (**c**) Process of the gas sensor stability test and dynamic response.

An indoor environment dynamic response and stability test and an actual environment test were designed for the sensor. The gas concentration signal is processed by the multi-channel data acquisition system and sent to the computer. Figure 8 illustrates the process of the gas sensor test and calibration. The gas sensor is enclosed in the gas chamber with a plastic inlet and outlet. The sealed chamber is shown in Figure 8 (a,b). A computer controls the multi-channel data acquisition system, the mass spectrometer, and a flow meter; simultaneously, it carries out software processing, calculation, flow control, and mass spectrometry, which is used to calibrate the sensors. During the experiment, the gas sensor was used to test ethylene, methane, dimethyl ether, and other gases. Figure 8(c) shows the process of gas sensor stability test and dynamic response.

**Figure 9.**
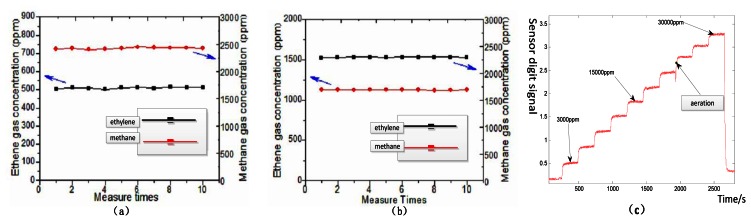
(**a**)Results of sensor repeatability test (**b**) Results of sensor stability test (**c**) Dynamic response of IR detector to gas concentration in the experimental test.

To evaluate a given gas sensor, we carried out repeatability and stability testing. The output shows the repeatability and stability gas response (Figure 9(a,b)). We increased the gas concentration by 3,000 ppm every half hour. Through the computer acquisition and processing, we obtained results as shown in Figure 9 (c). Figure 9 (a) shows the response of an IR detector to gas concentrations in the experimental test, as well as the gas sensor output in response to ethylene, methane, and dimethyl ether in the same circuit. With separate continuous measurements of ethylene and methane standard gases, 10 measurements were taken at 30-min intervals. The measurements were used to test the stability of the system. For ethylene, the relative error was 1.53% and the standard deviation was 0.0082; for methane, the relative error was 0.854% and the standard deviation was 0.0084. Therefore, the system displayed good reliability and stability. The indoor dynamic response and results of the sensor stability test are shown in Figure 9.

**Figure 10.**
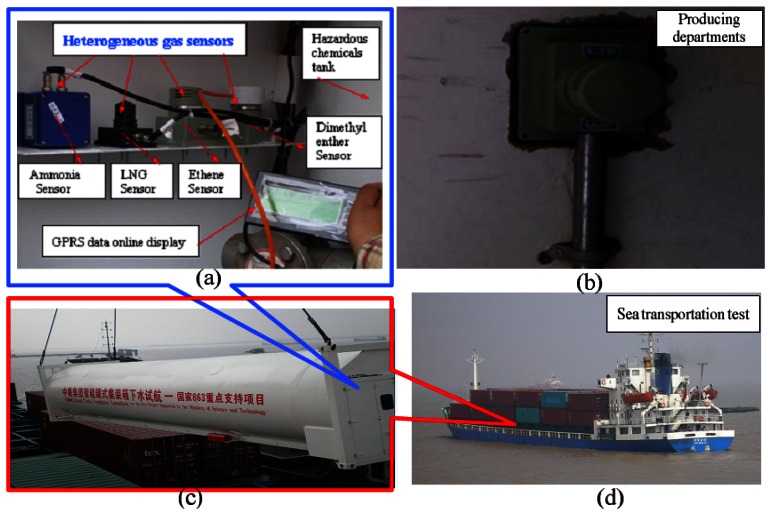
(**a**) Sensor system installed at the back of a transportation container; (**b**) Sensor system installed in a production plant; (**c**) Container of hazardous chemicals; (**d**) Tanker for Marine test.

To test the sensor outdoors in an on-site inspection loading environment, we used the main sensor for transportation and for safety in a production plant. At the same time, we installed a sensor system in the back of a container for the marine test. Figure 10 (a,c,d) shows the complete operation. To verify the feasibility of the sensor, the sensors are installed inside the production places and the tank container, and water is discharged into the tank (to simulate the transportation of hazardous chemicals), with different gas cylinders mounted in the vehicle in order to simulate a gas leak in the transportation process.

**Figure 11.**
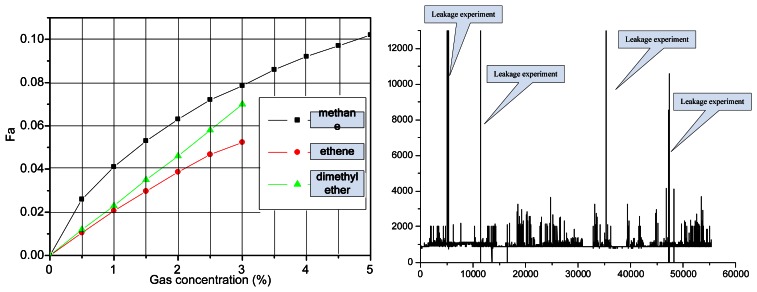
(**a**) Three types of gas sensor response relationships (ethylene, methane, dimethyl ether); (**b**) Experimental results for sea transportation of dimethyl ether.

Figure 11(a) shows three types of gas sensor response relationships (ethylene, methane, dimethyl ether), respectively, in the same design circuits and magnification conditions. Figure 11(b) shows the experiment result for sea transportation of dimethyl ether. From this figure, the sensor response output is inconsistent. Therefore, the filter in the 3.31μm wavelength region is used to detect most of the CH gas.

## 5. Conclusions

In this paper, we have described an optical gas leak sensor based on IR spectroscopy for detecting ethylene, dimethyl ether, and methane. The system was developed to prevent gas accidents in the processes of hazardous chemicals production, storage, transport, *etc*. Optical gas detection is superior to traditional detection methods in terms of accuracy, speed, and even security. Conventional devices for optical gas detection include a broadband source, a rotating chopper shutter, a narrow-band filter, a sample tube, and a detector. The sensor uses a miniature dual-channel detector, an electrical modulation source, and a miniature gas cell structure. The system is thus small, low-powered, and portable. The system has no moving parts, and is reliable and durable because of no chopper or mechanical modulators are required. By replacing the filter, we can also detect gases with different infrared absorption peaks, allowing for multi-gas detection with a single unit. Therefore, this sensor has a variety of potential industrial and military applications.

## Figures and Tables

**Table 1.** Some usefully specific parameters of gas detection filters.

**Gas**	**CWL/μm**	**Tolerance of CWL/nm**	**BW/nm**	**Tolerance of BW/nm**
Reference	3.95	±40	90	±20
CO_2_ standard	4.24	±40	180	±20
CO_2_narrow	4.24	±40	90	±20
CO_2_ high AOL	4.27	±30	170	±20
CO_2_ long path	4.45	±20	60	±20
HC	3.40	±30	120	±20
CO	4.66	±40	180	±20
NO_X_	5.30	±40	180	±20
SO_2_	7.30	±40	200	±30
